# CAR-T Cells and Oncolytic Viruses: Joining Forces to Overcome the Solid Tumor Challenge

**DOI:** 10.3389/fimmu.2018.02460

**Published:** 2018-10-23

**Authors:** Sonia Guedan, Ramon Alemany

**Affiliations:** ^1^Department of Hematology and Oncology, Hospital Clinic, Institut d'Investigacions Biomèdiques August Pi i Sunyer, Barcelona, Spain; ^2^ProCure Program, IDIBELL-Institut Catala d'Oncologia, L'Hospitalet de Llobregat, Spain

**Keywords:** chimeric antigen receptors (CAR), oncolytic viruses, solid tumors, immunotherapy, immunosuppressive tumor microenvironment, adoptive cell transfer (ACT)

## Abstract

Adoptive transfer of chimeric antigen receptor (CAR)-modified T cells has resulted in unprecedented rates of long-lasting complete responses in patients with leukemia and lymphoma. However, despite the impressive results in patients with hematologic malignancies, CAR-T cells have showed limited effect against solid cancers. New approaches will need to simultaneously overcome the multiple challenges that CAR-T cells encounter in solid tumors, including the immunosuppressive tumor microenvironment and heterogeneity of antigen expression. Oncolytic viruses are lytic and immunogenic anti-cancer agents with the potential to synergize with CAR-T cells for the treatment of solid tumors. In addition, viruses can be further modified to deliver therapeutic transgenes selectively to the tumor microenvironment, which could enhance the effector functions of tumor-specific T cells. This review summarizes the major limitations of CAR-T cells in solid tumors and discusses the potential role for oncolytic viruses as partners for CAR-T cells in the fight against cancer.

## Introduction

The recent approval by the US Food and Drug Administration (FDA) of two different CAR-T cell therapies for the treatment of leukemia and lymphoma represents a landmark in the development of cancer immunotherapies. Together with immune checkpoint blockade therapy (1), CAR-T cells are revolutionizing the field of cancer therapy, providing hope for a cure in patients with previously refractory cancers (2–8). However, despite the stunning results of CAR-T cells in patients with hematologic malignancies, this approach has shown little effect in patients with solid tumors. Recent clinical trials demonstrated that CAR-T cells are able to infiltrate the tumor mass and exert antigen-directed activity (9–12). However, with rare exceptions (13, 14), observed responses in patients with solid tumors have been minor and transient.

In order to induce complete responses in patients with solid tumors, CAR-T cells need to overcome several barriers. First, CAR-T cells must traffic from the blood into the tumor, infiltrate the tumor mass, and be able to survive and maintain their effector functions in a tumor microenvironment that is highly immunosuppressed and enriched in stroma. Then, CAR-T cells need to eliminate the totality of the cancer cells, which is extremely difficult due to the heterogeneity of antigen expression in cancer cells and the intrinsic plasticity of tumors that may lead to tumor escape (15, 16). Finally, CAR-T cells are living drugs that can lead to dramatic antitumor responses but can also induce significant toxicities (17–19). New approaches to enhance therapeutic outcome in patients with solid tumors must therefore focus on enhancing potency without increasing toxicity.

Rapid advances in synthetic biology, T cell immunology, and gene editing have fueled the design of next generation CAR-T cells with the potential to overcome some of the hurdles encounter in solid tumors (20). However, it is unlikely that CAR-T cell therapy alone will be sufficient to induce complete responses in the majority of cancers. Combining CAR-T cells with other cancer treatments that have different mechanisms of action and the potential to synergize with T cells may reduce tumor escape and increase the success rates of CAR-T cell therapy. As novel therapies emerge, rational combinations will need to be tested based on an understanding of the mechanisms underlaying tumor resistance to CAR-T cells.

Oncolytic virotherapy is a therapeutic approach to treat cancer that uses native or genetically modified viruses that selectively replicate within cancer cells (21). The field of oncolytic virotherapy has gained renewed attention after the FDA approval of Talimogene laherparepvec (T-VEC), an oncolytic herpes simplex virus type 1 (HSV-1) modified to express GM-CSF (22), and the recent reports of high response rates obtained in patients with advanced melanoma when combining T-VEC with checkpoint blockade (23, 24). Oncolytic viruses (OV) mediate their antitumor effect through a dual mechanism of action, including a direct lytic effect on tumor cells and the induction of anti-cancer adaptive immunity (25, 26). Moreover, OV can be further modified to selectively deliver therapeutic transgenes to the tumor microenvironment to enhance their antitumor potency or boost an antitumor immune response (27). All these characteristics make OV excellent potential partners to synergize with emerging immunotherapies, and several combinatorial approaches are being currently tested in preclinical and clinical trials (26, 28).

This review provides an overview of current barriers that CAR-T cells encounter in solid tumors, summarizes the advances in the field of OV and discusses the preclinical and clinical data that support the clinical testing of OV in combination with CAR-T cells to overcome the solid tumor challenge.

## CAR-T cells in solid tumors: challenges and limitations

While most of the early trials of CAR-T cells for solid tumors resulted in poor therapeutic outcomes, some case reports of dramatic clinical responses with manageable therapy-related toxic effects provide clear reasons for optimism (13, 14). Recent reports with second generation CAR-T cells suggest that CAR-T cells can traffic, persist, and proliferate in the tumor (9, 10). Moreover, evidence of transient antitumor activity has been observed in patients with difficult-to-treat tumors, such as glioblastoma (13), neuroblastoma (14), pancreatic cancer (12), and sarcoma. Here, we summarize the lessons learned in these clinical trials and discuss the hurdles that CAR-T cells must overcome for effective therapy, focusing on those challenges that OV may help to address.

### Trafficking, proliferation, and persistence

The ability of tumor-specific T cells to traffic to the tumor, proliferate, and persist is considered critical to achieve an effective anti-tumor response (14, 29, 30). While T cells can actively traffic to sites of disease, often tumors present low levels of inflammation and lack of the chemokines required for migration. Also, physical barriers, such as aberrant vasculature, increased stromal stiffness and high interstitial pressure, may impair T-cell infiltration. Once in the tumor, CAR-T cells must efficiently proliferate, and persist until the entirety of the tumor is eliminated. However, T-cell proliferation and persistence are often hampered due to T-cell intrinsic (T-cell fitness) or extrinsic factors (tumor microenvironment). The requirements for proliferation and persistence can be relaxed in some instances if regional delivery and redosing of CAR-T cells is a therapeutic option (31). For example, in a recent clinical trial, multiple intracranial injections of CAR-T targeting IL13Rα2 mediated a transient complete response in a patient with glioblastoma (13). In this patient, two intracranial CAR-T cell delivery routes were tested: intracavitary and intraventricular. While intracavitary therapy was only able to control growth of the local tumor, intraventricular therapy resulted in a dramatic reduction in the size of all intracranial and spinal tumors. These results highlight the importance of trafficking and administration route to achieve the optimal tumor responses. Developing strategies to enhance trafficking and persistence to increase the therapeutic CAR-T cell input in the tumor would represent a vertical advance in the field.

### Tumor immunosuppression

On arrival to the tumor, CAR-T cells encounter an immunosuppressive environment that prevents T-cells from reaching their full therapeutic potential. The main barriers that CAR-T cells need to overcome once in the tumor include: (i) suppression by immunoregulatory cells, including myeloid-derived suppressor cells (MDSC), tumor associated macrophages and neutrophils, and regulatory T cells; (ii) presence of an array of immunosuppressive molecules, such as IL-10, TGF-β, PD-L1, IDO and arginase-1, and (iii) microenvironment factors, such as hypoxia, low pH and nutritional depletion. These conditions, together with chronic antigen exposure, can lead T-cells to distinct stages of functional dysfunction (32–34). Moreover, the stromal microenvironment can actively exclude T cells from the vicinity of cancer cells (35). Finally, a recent clinical report suggests that the tumor microenvironment can become even more immunosuppressive after CAR-T cell activation within the tumor, probably due to an initial production of IFN-γ (10). Finding ways to prevent or reverse T-cell dysfunction by reverting tumor immunosuppression will be key to improving treatment.

### Tumor escape by loss or heterogeneity of antigen expression

One of the main limitations in the treatment of solid tumors with CAR-T cells is the absence of cancer-restricted antigens that are uniformly expressed in tumor cells and absent in essential organs. Solid tumors exhibit heterogeneity of antigen expression with regards to intensity and distribution. Tumor escape due to heterogeneity or loss of antigen expression is an emerging threat to CAR-T cells, as it can result in overgrowth of target-deficient tumor cells that are invisible to CAR-T cell therapy (36–38). Preclinical studies have demonstrated that tumor cells expressing high levels of the targeted antigen are preferentially eliminated by CAR-T cells, whereas those with the lowest expression may survive (39–41). Decreased expression of the targeted antigen after CAR-T cell therapy has been observed in several clinical trials, including those targeting Her2 (9), EGFRviii (10), IL13Rα2 (11), and mesothelin (12). These results demonstrate the potential of CAR-T cells to eliminate antigen-positive tumor cells, but also highlight the importance of designing new strategies to simultaneously target different antigens. Several groups are designing new CAR constructs able to target more than one antigen simultaneously (39, 42, 43). While reducing the risk of escape, these strategies may also result in increased on-target off-tumor reactivity, as most of the targeted antigens can be expressed in healthy tissue at low levels (17–19). An alternative approach would be to find strategies to activate an endogenous immune response that could partner with CAR-T cells to completely eliminate the tumor. Some reports suggest that CAR-T-cell mediated tumor destruction may lead to the release of other tumor antigens that are cross-presented in a process known as epitope spreading (44, 45). This observation requires further investigation, but it could explain how complete elimination of tumor lesions has been achieved even when the tumors did not uniformly express the target (13).

## Oncolytic viruses: lessons learned in clinical trials

To date, there are three viruses commercially available for the treatment of cancer: T-VEC approved in the USA, H101 approved in China and Rigvir approved in Latvia, Georgia and Armenia. Several other viruses are in clinical trials and may eventually join this short list of marketed viruses (46). Some of the lessons learned from clinical trials that will drive the design of future therapies include: (*a*) OV can induce a therapeutic benefit in cancer patients, including complete responses, in the absence of severe adverse effects (47–50). Interestingly, some of these complete responses are reached after the virus have been eliminated, suggesting that the complete elimination of the tumor may depend on the activation of an immune-mediated anti-tumor response (48). On line with this observation, a recent clinical trial reported that the overall survival among patients who received a chimeric poliovirus reached a plateau of 21% 1 year after treatment that was sustained for months (51). This plateau in long-term survival is similar to the one observed in Kaplan-Meier curves from cancer patients treated with other cancer immunotherapies and highlights the role of the immune system on the emergence of long-term survivors (52); (*b*) The antiviral immunity constitutes an obstacle against OV as it sequesters or neutralizes viral particles before they reach their target. A major question is how to deliver the virus to the tumor efficiently; (*c*) Virus replication has been detected in tumor biopsies a few days after treatment. However, the ability of OV to survive and spread through the tumor is limited by antiviral T cells (47, 48, 53); (*d*) Tumors treated with OV typically show increased immune cell infiltration, including activated macrophages and cytotoxic T-cells, and pro-inflammatory cytokines (47, 48, 53). Tumor-specific T cells have been detected after treatment with OV (53, 54). While the capacity of OV to expand neoantigen-specific T cells deserves further investigation, the potential of OV for combination with immunotherapies such as immune checkpoint inhibitors has been well-recognized (28, 55–58). Several clinical trials are currently testing the combination of OV with immune checkpoint therapy and initial reports showed promising results (23, 24).

## Oncolytic viruses: the ideal allies for CAR-T cells?

OV have the potential to synergize with CAR-T cells by helping them simultaneously overcome some of the multiple barriers found in solid tumors. First, viruses provide a danger signal that can revert tumor immunosuppression, which could facilitate CAR-T cell trafficking, proliferation, and persistence in the tumor microenvironment. Second, the direct lytic effect of OV on cancer cells results in tumor lysis and release of tumor-associated antigens (TAA), which can induce an anti-tumor adaptive response that could potentially mitigate tumor escape by antigen loss. Third, OV can be armed with therapeutic transgenes that could further enhance the effector functions of T cells. Here, we provide an overview of the biological properties of OV that may be considered when choosing a viral platform for combination with CAR-T cells, and we summarize the recent preclinical strategies that have been explored combining CAR-T cells and OV.

### Oncolytic viruses as immunotherapy agents

The immune system is well-equipped to mount an innate inflammatory response to viruses that eventually will induce the infiltration of effector T-cells. In particular, OV have pathogen-associated molecular patterns (PAMPS) detected by pattern-recognition receptors (PRRs) on tumor and epithelial cells as well as macrophages and dendritic cells (59). These PRRs induce danger associated molecular patterns (DAMPs) characteristic of an immunogenic cell death (60, 61). PRRs also signal through NF-kB to induce the expression of cytokines such as TNF-α and IL6, and through IFN Regulatory Factor (IRF) to induce type I interferons and activate caspase 1 that matures IL-1β (62). This pro-immune cytokine environment can facilitate the maturation and function of DC's, macrophages, and epithelial cells that can lead to the recruitment of neutrophils and natural killer (NK) cells, monocytes, and memory T-cells to the site of infection (63–65). Tumor cells dying due to the lytic activity of OV can release TAA. Activated DC's with their MHC loaded with virus and/or tumor epitopes can traffic to the draining lymph nodes to engage specific T-cells and stimulate their proliferation and circulation into the bloodstream. Chemokines of the infected tumors can induce integrin expression on these T-cells and selectin expression on endothelial cells to extravasate them. Under these conditions, T cells can be recruited efficiently to infected tumors, and as discussed above, increased T cell infiltration is generally detected in tumors of patients treated with OV therapy. Interestingly, viral infection has been shown to induce neoantigen-directed T cell responses (53, 54), which could synergize with CAR-T cells and virus-specific T cells to clear the tumor. A mayor limitation to study the impact of the immune-modulating effects of OV on CAR-T cell therapy is the lack of good animal models. However, it can be hypothesized that following the establishment of a more immunogenic intratumoral milieu, killing of target cells may be more efficient due to cooperation between the effector T-cells.

The ability of OV to induce an anti-tumor immune response is now considered a key mechanism of action to obtain long-term antitumor responses. Therefore, most of the current efforts directed at enhancing the therapeutic potential of OV are focused on improving their capacity to induce a systemic antitumor response.

### The oncolytic virus armamentarium

Multiple types of viruses are used in cancer virotherapy, each one of them with its unique properties (Table 1) (66). Here we discuss some of the different factors that should be considered when selecting an OV for combination with CAR-T cells. In general terms, viruses that replicate in the cytoplasm (RNA viruses) kill tumor cells faster than nuclear ones (DNA viruses) as they do not need to reach the nucleus of the infected cells. But for the same reason, they offer less opportunities for tumor-selective control. Tumor-selective replication of most oncolytic RNA viruses, such as reovirus, picornaviruses (Coxsackeivirus, Rigavirus), rhabdovirus (Vesiscular Stomatitis Virus [VSV], Maraba Virus), and paramixovirus (Measles Virus, Newcastle disease virus [NDV]), depends on defects of the interferon pathway in tumor cells. Because IFN induction is a central pathway in the innate response to viruses, which potentiates the adaptive T cell responses, the inflammatory response elicited with these viruses is expected to be lower. DNA viruses, such as adenoviruses, have slower replication cycles but are amenable to being controlled in the nucleus of the infected cells using tumor-selective promoters. The presence of an envelope also determines the oncolytic properties of a virus. Enveloped viruses (i.e., Measles virus, NDV, VSV, Herpes simplex virus, and Vaccinia virus) bud from cells and are less “lytic” than naked viruses. The envelope also contributes to the main clearance mechanisms in blood, with complement having a major role for enveloped viruses and antibodies for non-enveloped ones. Size is also an important parameter for the properties of OVs. The smaller the virus, the easier it will be for the virus to penetrate and diffuse throughout the tumor. But a larger virus with a larger genome allows the insertion of non-viral transgenes. Arming OV with therapeutic transgenes offer the opportunity to complement the OV in multiple ways. Among RNA viruses, VSV, Measles virus, and NDV can accept transgenes in contrast to picornaviruses and reoviruses, and for DNA viruses, Adenovirus, Herpes Simplex Virus and Vaccinia virus can be armed with transgenes in contrast to parvovirus. The list of genes that have been included in OV that could be potentially useful for combination with CAR-T cells is long and it has been reviewed recently (67). It includes, among others: (a) inducers of immunogenic cell death (68), (b) transgenes directed to modulate the immune system, such as cytokines (22, 69–71), chemokines (72, 73), co-stimulatory proteins (74–77), bispecific T-cell engagers (BiTEs) (78, 79), and immune checkpoint blockers (80–83), and (c) stroma-degrading proteins that could facilitate the spread of OV and T-cells within the tumors (84, 85). Comparing viruses and transgenes is a very challenging task given the limitations of preclinical immune competent mouse models, where many human viruses present defects in replication and tumors do not edit the immune system in a slow and progressive way as occurs in humans.

**Table 1:** Main groups of oncolytic viruses classified by taxonomy families.

**Virus family**	**Genome type**	**Genome size (Kb)**	**Replication site**	**Capsid**	**Mechanism of selectivity**	**Armed**	**Viruses in clinical development (or approved when indicated)**
**Retroviruses:**Murine leukemia virus	dsRNA (requires integration in cell genome as DNA)	8	Nucleus	Enveloped	Requires cell division to enter the nucleus	Transgenes	Toca-511
**Picornaviruses:** Coxsackievirus A21 ECHO-7 Seneca virus Poliovirus	ssRNA	7.5	Cytoplasm	Naked	IFN sensitive (IFN inhibits protein translation and virus replication but in tumor cells this protein translational block is impaired) Receptor tropism: CVA21 infects by ICAM-1 as primary receptor and DAF as coreceptor.	miRNAs Only small insertions (300bp)	CAVATAK Rigvir (Approved) SVV-001 PVSRIPO
**Rhabdoviruses:** Vesicular stomatitis virus (VSV) Maraba	ssRNA	15	Cytoplasm	Enveloped	IFN sensitive M protein mutations increase IFN sensitivity	Transgenes	VSV-IFNβ VSV-IFNβ-NIS Maraba MG1-MAGEA3.
**Paramyxoviruses:** Meales Newcastle disease virus (avian virus)	ssRNA	16-20	Cytoplasm	Enveloped	IFN sensitive Edmonton Measles strain infects via CD46 and SLAMs found in B and T lymphocytes. Appropriate for myeloma.	Transgenes	MV-CEA MV-NIS NDV PV701 NDV MEDI5395
**Reoviruses:** Mammalian orthoreovirus Type 3 Dearing	dsRNA (in fragments or segments)	22-27	Cytoplasm	Naked	IFN sensitive	No	Reolysin
**Parvoviruses:**H1 autonomous rat parvovirus	ssDNA	5	Nucleus	Naked	Unknown (active metabolic and regulatory pathways allow replication of this rat virus in human tumor cells).	No	H1-PV Parvoryx
**Adenoviruses**	dsDNA	36	Nucleus	naked	RB pathway if E1a binding site to pRB is deleted Control of gene expression with tumor selective promoters.	Transgenes	H101 (approved) CG0070 LOAd703 DNX-2401 Telomelysin ColoAd1 ONCOS-102 VCN-01
**Herpesviruses**	dsDNA	150	Nucleus	Enveloped	Becomes IFN sensitive if viral gamma 34.5 gene is deleted. Depends in high nucleotide metabolism if viral Thymidine Kinase gene is deleted. Control of gene expression with tumor selective promoters.	Transgenes	Tvec (Imlygic) (Approved) HSV1716 G207 RP1
**Poxviruses:** Vaccinia Virus (VV) Mixoma Virus (of rabbits)	dsDNA	190	Cytoplasm	Enveloped	Becomes IFN sensitive if viral B18R gene is deleted. Depends in high nucleotide metabolism if viral Thymidine Kinase or Ribonucleotide reductase genes are deleted. Depends on EGF-R pathway if VGF gene is deleted.	Transgenes	JX-594 TG6002 GL-ONC1 GLV-1h68

*Key parameters for oncolysis are shown. Some examples of viruses in clinical development are included for each virus family. Top RNA viruses, bottom DNA viruses. Ordered by genome size*.

### Combining CAR-T cells and oncolytic viruses for the treatment of solid tumors

**Figure 1:**
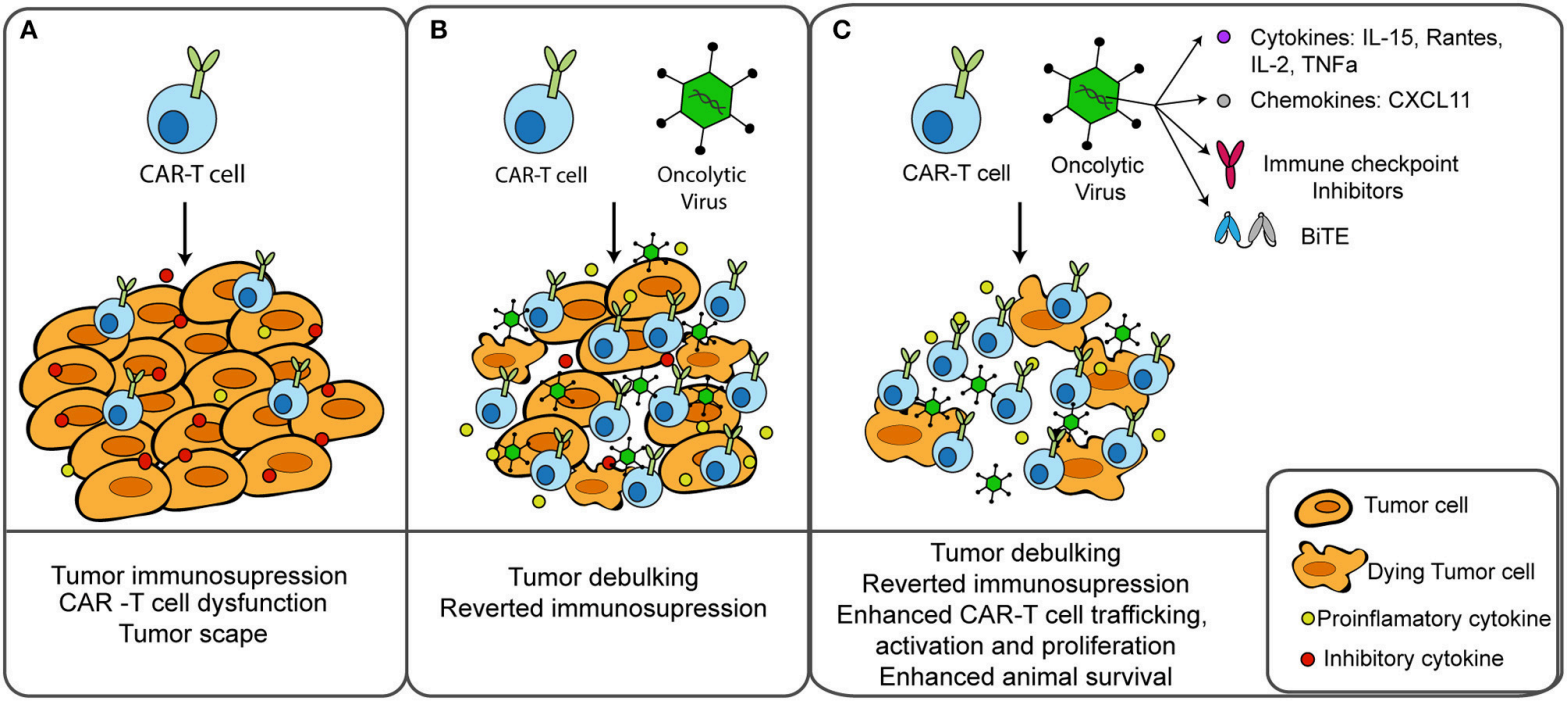
Combination of CAR-T cells and oncolytic virus for the treatment of solid tumors. **(A)** CAR-T cells find several obstacles in solid tumors, including an immunosuppressive environment that can lead to T cell dysfunction and treatment failure. **(B)** Cancer treatment with oncolytic viruses prior to CAR-T cell therapy results in tumor debulking, immunogenic cell death and reverted tumor immunosuppression. **(C)** Oncolytic viruses can be genetically modified to deliver therapeutic transgenes into the tumor microenvironment to enhance T-cell effector functions. Preclinical studies combining CAR-T cells with oncolytic viruses armed with cytokines, chemokines, BiTEs, or immune checkpoint inhibitors resulted in enhanced therapeutic outcomes.

At a preclinical level, several groups have started to test different transgene-armed OV in combination with CAR-T cells (Figure 1). Most of these works assessed the antitumor effects of these therapies in NOD scid gamma (NSG) mice, a mouse strain that is completely deficient in adaptive immunity and severely deficient in innate immunity (86). NSG mice allow the engraftment and persistence of adoptively transferred CAR-T cells, and human tumor xenografts allow the replication of the virus and the delivery of the transgene. Therefore, these studies gave important insights in the antitumor effects of combining CAR-T cells with oncolysis and transgene delivery. An important limitation is that the capacity of OV to induce anti-tumor immunity cannot be assessed using these tumor xenografts.

Oncolytic adenoviruses modified to express IL-15 and RANTES (87) or IL-2 and TNF-α (88) have been shown to increase the accumulation and survival of CAR-T cells in the tumor microenvironment. Similarly, with the goal of enhancing the intra-tumoral trafficking of CAR-T cells, a vaccinia virus expressing CXCL11, a CXCR3 ligand, was used to attract effector cells following transfer (89). Another report demonstrated that expression by an oncolytic adenovirus of a BiTE targeting a second tumor antigen could address heterogeneity of antigen expression (40). Combination of a preparation of CAR-T cells with the OV-BiTE induced activation of T cells in the absence of the CAR-targeted antigen or lack of CAR expression (i.e., non-transduced T cell population). In a slightly different approach, combination of an oncolytic adenovirus with a helper-dependent adenovirus expressing a PD-L1 blocking mini-antibody was used to revert T cell dysfunction by preventing PD1:PDL1 interaction (90). Co-expression of IL12p70 and PD-L1 further augmented the therapeutic efficacy of the combination (91). As expected, all these combinations of CAR-T cells and armed-OV resulted in enhanced tumor control and prolonged survival when compared to each agent as monotherapy. An interesting finding by Watanabe et al. is that CAR-T cells as monotherapy failed to control the growth of the primary tumor, while OV could suppress the progression of the primary tumor but mice died from metastatic disease. Combination of CAR-T cells with an OV armed with IL-2 and TNF-a was able to control both the primary tumor and tumor metastasis (88).

Finally, in a totally different and very preliminary approach, CAR-T cells have been used to deliver OV to the tumor (92). Circulating cells such as lymphocytes, monocytes, erythrocytes, or even platelets can bind viruses and have shown tumor-targeting properties (93–96). Loading OV onto tumor-specific T cells (by adhesion to the T-cell surface) can protect the virus from neutralizing antibodies while retaining its antitumor efficacy after release in the tumor microenvironment (96). OV-tumor delivery by CAR-T cells could enhance virus delivery to the tumor and subsequent oncolysis could attract more CAR-T cells, establishing a positive feedback loop.

## Remaining questions and future directions

With such a variety of oncolytic viruses it is hard to know which one will be best suited for combination with CAR-T cells. In practical terms, it is difficult to envisage a virus commercially developed solely for the combination with CAR-T cells. Therefore, marketed viruses or viruses under clinical investigation are expected to be the first ones to be used in the clinic in combination with CAR-T cells.

While the general value of the virus to attract T-cells to the tumor is widely accepted (53, 97), practical questions on best delivery routes and dosing schedules are more difficult to predict. Intratumoral administration of the OV provides larger amounts of virus in the injected tumors, but it is technically challenging for visceral tumors or metastases, and non-injected tumor lesions will be less likely to get any virus to change the immunosuppressive microenvironment. Systemic intravenous administration is easier to perform and potentially useful to reach all metastases, but efficient neutralization of the virus in the bloodstream, especially with high titers of neutralizing antibodies raised after the first virus administration, will impose a barrier for repeated delivery. The immune response to the virus may also be very different if the virus is injected intratumorally or systemically. Usually vaccination immunization is performed subcutaneously or intramuscularly as the immune system does not respond aggressively to systemic pathogens, partly due to a lower inflammatory response of liver Kupffer cells compared to tissue-resident dendritic cells and the tolerogenic nature of the liver (98). Therefore, the immune response elicited by an OV replicating in a tumor may be tamed or modulated when the virus has been detected systemically. Timing of the virus and CAR-T cells can also impact the outcome. In principle, the virus should go first to change the immune suppressive tumor microenviroment, induce a direct lytic effect on tumor cells, and create a more appropriate environment that attracts the CAR-T cells. Patient preconditioning should also be considered prior to therapy. Although the immunostimulatory environment generated by the virus may bypass the need to lymphodeplete the patient to promote CAR-T cell expansion, lymphodepletion could still be a good approach to foster virus replication and persistence in the tumor while providing an advantage to the co-administered CAR-T cells (4, 99, 100).

Oncolytic viruses offer a strong inflammatory self-amplification oncolytic mechanism of action that can also result in the release of TAA. However, the ability of OV to induce an anti-tumor immune response is not well-understood. Given the large number of viral non-self-peptides after treatment with OV, it is likely that immune responses to the viral epitopes will dominate the response in a mixture with tumor neoantigens (101–103). New strategies to increase the immunogenicity of tumor epitopes and reduce the immunodominance of viral antigens are needed to promote epitope spreading (104).

Finally, T cells could also be manipulated to become a better partner for oncolytic viruses. Virus-specific T cells have been used as a platform for CAR expression (105). Virus-specific CAR-T cells retain the ability to recognize both virus-infected and tumor targets through their native and chimeric receptors, respectively. Thus, these T-cells could be ideal for a combined treatment with OV, as the presence of the virus could boost the amplification of CAR-T cells in the tumor. A drawback of this approach is that a faster clearance of the OV will occur.

## Author contributions

SG and RA conceptualized, wrote, and edited the manuscript.

### Conflict of interest statement

The authors declare that the research was conducted in the absence of any commercial or financial relationships that could be construed as a potential conflict of interest.
